# The Heart and Its Difficulties

**Published:** 1899-01-14

**Authors:** 


### THE HEART AND ITS DIFFICULTIES.

THE admirable little address given by Sir William Broadbent at the Medical Society last Monday on "The Conduct of the Heart in the Face of Difficulties" served to emphasise a point in treatment which is too often forgotten, namely, that it is generally far better to remove the difficulty than to urge on the heart to greater efforts.

The difficulties which the heart meets with in the performance of its work vary much both in nature and degree. Sometimes they arise from mechanical interference with its action, as when they are caused by effusion in the pleura, or by pneumo-thorax, and especially by distension of the stomach or colon; sometimes they arise from increased arterial tension, and sometimes, as in valvular disease, the work of the heart is increased in consequence of its own imperfections. But whatever may be the difficulty, the important thing is the conduct of the heart in the face of it. Sometimes the heart meets its difficulties bravely, puts on muscle, grows in strength, and so completely overcomes the obstruction that "compensation," as we call it, is complete; sometimes, like many good people in this world, it labours hard, but its efforts lead only to failure, the heart grows thin and weak, and dilatation is the beginning of the end; while in some cases, again, like other people who are not so good, the heart is restive and irritable from the beginning, making much of little troubles, palpitating, becoming irregular, and even stopping altogether, just because of some little shock which other hearts would take no heed of. While, then, the conduct of the heart is no measure of the gravity of the difficulty by which its action is disturbed, on the other hand the importance and the prognosis of the disease (so far as disease is shown by physical signs) cannot be rightly judged of without due consideration being given to the manner in which the heart conducts itself in the face of the difficulties in its path.

Sir William Broadbent pointed out that, so far as the patient's misery is concerned, disorder of the stomach or of the colon may accurately simulate the most serious forms of cardiac disease, while even in organic heart disease much of the distress is often due to such conditions, acting partly in a mechanical manner and partly through the nervous system. The neuro-muscular apparatus of the heart in fact partakes largely of the character of the nervous system of the individual, and thus when the nervous centres are sensitive and easily disturbed, the symptoms of severe heart disease may be set up by apparently trivial causes.

So far as to how the heart conducts itself. But there is another side of the question, namely, how should the physician conduct the heart through its difficulties? Take the case of a small mitral regurgitation. Are we, when we discover this little leak, to frighten the patient out of his wits, and tell him to avoid exertion and live on one floor? Let us first see how the heart is facing its difficulties. If, notwithstanding the defective valve, there is no indication of any back pressure, no enlargement of the right side of the heart, no increase of tension in the pulmonary artery, no disturbance of the heart's action, no palpitation, and no breathlessness on exertion, that patient, for all his "leak," has not got a diseased heart, and the less said about it the better. But things may not be quite so favourable. There may be signs of increased tension in the pulmonary artery, there may be some enlargement of the ventricles, but if there are no other signs of cardiac disorder, no palpitation and no breathlessness, if, in fact, compensation is complete, the patient again must not be treated as if he had a diseased heart. So long as compensation is maintained he may do much as others do; he may shoot and hunt, but the line should be drawn somewhat short of football. He should, however, be warned that the compensation may give way; but in the treatment of the case it must be remembered that the compensation is far more likely to be durable under the influence of exercise and fresh air than under care and coddling. But if compensation is insufficient, and if on exertion breathlessness is felt, then we have disease, and the patient must be treated for his malady.

When, then, have we to do with actual disease. Are we to endeavour to stimulate the heart? Certainly, sometimes; but first let us, as far as we may, remove or lessen the difficulty with which it is struggling. The loaded stomach or distended colon may be relieved; the arterial tension may be lessened; and where a dilated heart is struggling against obstruction, its efforts being paralysed by its great load of blood, the load may be lightened by venesection. Nothing approaches so nearly to a therapeutic miracle as the results of blood-letting in a case of dilated right ventricle from pulmonary obstruction. Why is this therapeutic miracle not more often performed? Sir William Broadbent says that the only instrument he habitually carries is a lancet, which he always has with him in his purse; his reason being that, when he advises bleeding, he often finds that the practitioner whom he meets has never bled a patient in his life, and sometime has not even seen it done. So the consultant has to perform the miracle himself, and gets the kudos. This is a little lesson which should be taken well to heart.

